# The clinical implications of FDG-PET/CT differ according to histology in advanced gastric cancer

**DOI:** 10.1007/s10120-018-0847-5

**Published:** 2018-06-09

**Authors:** Hong Jae Chon, Chan Kim, Arthur Cho, Yoo Min Kim, Su Jin Jang, Bo Ok Kim, Chan Hyuk Park, Woo Jin Hyung, Joong Bae Ahn, Sung Hoon Noh, Mijin Yun, Sun Young Rha

**Affiliations:** 1 Medical Oncology, CHA Bundang Medical Center, CHA University, Seongnam, South Korea; 2 Yonsei Graduate School, Yonsei University College of Medicine, Seoul, South Korea; 3 Department of Nuclear Medicine, Yonsei University College of Medicine, 50 Yonsei-ro, Seodaemun-ku, Seoul, 120-752 South Korea; 4 Department of Surgery, CHA Bundang Medical Center, CHA University, Seongnam, South Korea; 5 Department of Nuclear Medicine, CHA Bundang Medical Center, CHA University, Seongnam, South Korea; 6 Biostatistics Collaboration Unit, Department of Research Affairs, Yonsei University College of Medicine, Seoul, South Korea; 7 Department of Internal Medicine, Hanyang University Guri Hospital, Hanyang University College of Medicine, Guri, South Korea; 8 Department of Surgery, Yonsei University College of Medicine, Seoul, South Korea; 9 Department of Internal Medicine, Yonsei University College of Medicine, 50 Yonsei-ro, Seodaemun-ku, Seoul, 120-752 South Korea

**Keywords:** Advanced gastric cancer, PET/CT, Prognostic impact, Signet ring cell carcinoma, Diffuse type

## Abstract

**Background:**

The prognostic impact of preoperative ^18^F-FDG PET/CT in advanced gastric cancer (AGC) remains a matter of debate. This study aims to evaluate the prognostic impact of SUV_max_ in preoperative ^18^F-FDG PET/CT of AGC according to histologic subtype, with a focus on the differences between tubular adenocarcinoma and signet ring cell (SRC) carcinoma.

**Methods:**

As a discovery set, a total of 727 AGC patients from prospective database were analyzed according to histologic subtype with Cox proportional hazard model and p-spline curves. In addition, another 173 patients from an independent institution was assessed as an external validation set.

**Results:**

In multivariate analysis, high SUV_max_ in preoperative ^18^F-FDG PET/CT of AGC was negatively correlated with disease-free survival (DFS) and overall survival (OS) in patients with diffuse type (DFS: HR 2.17, *P* < 0.001; OS: HR 2.47, *P* < 0.001) or SRC histology (DFS: HR 2.26, *P* = 0.005; OS: HR 2.61, *P* = 0.003). This negative prognostic impact was not observed in patients with intestinal type or well or moderately differentiated histology. These findings have been consistently confirmed in a validation set. The p-spline curves also showed a gradual increase in log HR as SUV_max_ rises only for SRC histology and for diffuse-type AGC. Finally, a novel predictive model for recurrence of AGC with diffuse type or SRC histology was generated and validated based on the preoperative SUV_max_.

**Conclusions:**

Preoperative high SUV_max_ of AGC is a poor prognostic factor in those with diffuse type or SRC histology. This study is the first to demonstrate the differential prognostic impact of preoperative PET/CT SUV_max_ in AGC according to histologic subtype and provide a clue to explain previous discrepancies in the prognostic impact of preoperative PET/CT in AGC. Prospective studies are required to validate the role of preoperative SUV_max_ in AGC.

**Electronic supplementary material:**

The online version of this article (10.1007/s10120-018-0847-5) contains supplementary material, which is available to authorized users.

## Introduction

F-18 fluoro-2-deoxyglucose positron emission tomography/computed tomography (^18^F-FDG PET/CT) has become an indispensable method for the diagnosis, staging, and response evaluation of many malignancies [1–3]. In gastric cancer (GC), ^18^F-FDG PET/CT is a useful tool for the diagnosis of recurrent disease after curative surgery [4–7]. However, the role of preoperative ^18^F-FDG PET/CT is not yet fully established. While the National Comprehensive Cancer Network guidelines recommend the use of preoperative ^18^F-FDG PET/CT in GC patients to rule out distant metastasis, the prognostic impact of preoperative ^18^F-FDG PET/CT remains a matter of debate. Several reports suggest a potential prognostic role for preoperative ^18^F-FDG PET/CT, while others argue against this [8–10]. These discrepancies may be due in part to small sample sizes and heterogeneous patient populations in different studies. Notably, ^18^F-FDG PET/CT has low sensitivity in detecting early GC (EGC) and signet ring cell (SRC) GC, but many previous studies overlooked this, including heterogeneous populations in the studies and analyzing the clinical characteristics of the population as a whole. Because SRC GC is a unique histologic subtype of GC with a distinct tumor biology and bioenergetics, it should be analyzed separately [11–14].

The present study evaluated the prognostic impact of SUV_max_ in preoperative ^18^F-FDG PET/CT of advanced GC (AGC) according to histologic subtype, with a focus on the differences between tubular adenocarcinoma and SRC carcinoma.

## Materials and methods

### Patient selection

Between January 2006 and December 2013, patients with GC who underwent ^18^F-FDG-PET/CT and subsequent curative surgical resection at Yonsei Cancer Center, Severance Hospital, Seoul, Korea, were enrolled in the study. A predesigned data collection format was utilized to extract data from a prospectively maintained database. The main eligibility criteria were as follows: (1) pathologically confirmed AGC of tubular adenocarcinoma or SRC-histologic subtype; (2) available documented information regarding the primary tumor site, postoperative pathological stage, surgery, recurrence, and survival; and (3) patients who received curative resection including those who presented with enlarged paraaortic lymph nodes having radical surgical resection with a curative aim accompanied by paraaortic lymph node dissection. The main exclusion criteria were as follows: (1) patients with EGC; (2) patients who received neoadjuvant chemo- or radio-therapy; and (3) patients with multiple primary cancers. After applying these criteria, 727 of the original 1605 patients were included in the final analysis (Fig. S1). The pathological stage was classified according to the American Joint Committee on Cancer (AJCC) staging manual (7th edition). This study was approved by the Institutional Review Board of Severance Hospital (#2015-1751-001). For a validation cohort, AGC patients who underwent preoperative ^18^F-FDG-PET/CT and curative surgical resection at CHA Bundang Medical Center, Seongnam, Korea, between March 2007 and February 2014, were enrolled in the study. Data acquisition and analysis were adopted identically as in the aforementioned institution.

The WHO and the Lauren classifications were used for the histopathological evaluation of surgical specimens. Tubular adenocarcinoma was additionally classified as being well, moderately, or poorly differentiated according to the WHO classification. Accordingly, we divided the patients into three groups for further analyses: well or moderately differentiated (WMD), poorly differentiated (PD), and SRC. In terms of the Lauren classification, the tumors were classified as intestinal, diffuse, or mixed type.

### ^18^F-FDG PET/CT and image analyses

All ^18^F-FDG PET/CT were performed with either the Discovery STe PET/CT (GE Healthcare, Milwaukee, WI, USA) or the Biograph TruePoint 40 PET/CT (Siemens Healthcare, Erlangen, Germany). All patients fasted for at least 6 h before the scan, and the glucose level in the peripheral blood of all patients was confirmed to be 140 mg/dL or less before ^18^F-FDG injection. Approximately, 5.5 MBq ^18^F-FDG/kg body weight was administered intravenously 1 hour before image acquisition. After the initial low-dose computed tomography (CT) (Discovery STe: 30 mA, 140 kVp, Biograph TruePoint: 36 mA, 120 kVp), standard PET/CT imaging was performed from the neck to the proximal thighs with acquisition times of 2.5 min/bed position for the Biograph Truepoint 40 PET/CT and 3 min/bed position for the Discovery STe scanner in three-dimensional mode. Images were then reconstructed using ordered subset expectation maximization (2 iterations, 20 subsets).

The images were retrospectively reviewed on a GE AW 4.0 workstation by two experienced nuclear medicine specialists (A.C. and M.Y.) who were unaware of the patients’ clinical information, except for the diagnosis of GC. The evaluation of ^18^F-FDG PET/CT images was performed in two steps. First, ^18^F-FDG PET/CT images of all patients were visually assessed and the patients were classified as positive or negative with respect to ^18^F-FDG uptake in the primary tumor. Lesions showing focally increased ^18^F-FDG uptake that exceeded the uptake in the surrounding stomach wall and corresponding cancer lesions as observed by contrast-enhanced CT images and gastroduodenoscopy were classified as positive ^18^F-FDG uptake. Focally or diffusely increased ^18^F-FDG that was indistinguishable from physiological gastric wall uptake was judged to be negative ^18^F-FDG uptake. After the visual assessment, the maximum standardized uptake value (SUV_max_) of the primary lesion was obtained and recorded for semi-quantitative analysis.

For the validation cohort, the PET/CT imaging for 173 AGC patients from CHA Bundang Medical Center was performed with Biograph mCT 128 scanner (Siemens Medical Solutions, Knoxville, TN, USA). Initial low-dose CT scans for attenuation correction (120 kV, 120 mA, 3 mm section width, 3 mm collimation) and PET/CT scans of same area with three-dimensional mode were acquired consecutively. Images were reconstructed on 400 × 400 matrices using the TrueX algorithm plus time-of-flight (TOF) reconstruction and analyzed using a dedicated workstation and software (Syngo.via, Siemens Medical Solutions, Knoxville, TN, USA). Unless otherwise stated, all other methods applied for image acquisition and data analysis were adopted identically as in the aforementioned institution.

### Statistical analysis

The cut-off date was December 31, 2015. The mean SUV_max_ was compared according to the patients’ basic demographic and clinical characteristics using independent sample t tests or analysis of variance. For pairwise comparisons of each level of categorical variables, the statistical significance was adjusted for inflated type I errors from multiple comparisons using the Bonferroni method.

Relapse-free survival (RFS) was measured from the time of surgery to initial tumor relapse (either local or distant) or death from any cause, and overall survival (OS) was calculated as the time from surgery to death from any cause or to the last follow-up date. Survival outcomes of the group with high SUV_max_ were compared with survival outcomes of the group with low SUV_max_ based on the median SUV_max_ for each histologic subgroup using the log-rank test. The Cox proportional hazards model was used for multivariable analysis of prognostic factors, including age at diagnosis, sex, stage, and PET/CT SUV_max_. To determine additional associations between SUV_max_ and survival outcomes, we examined the Cox regression model using the penalized spline smoothing method as described previously [15]. The performance of prognostic models was measured by Harrell’s c-index. We assessed model calibration by plotting the model-predicted- and actual observed 3- and 5-year RFS probabilities as calculated using the Kaplan–Meier method. The bootstrapping method with 1000 re-samples was used for adjusting bias and checking the interval validation. Statistical significance was set as *P* < 0.05 for all analyses. All statistical analyses were performed using SPSS version 20.0 (SPSS, Chicago, IL, USA), SAS version 9.4 (SAS Institute Inc., Cary, NC, USA), and R package, version 3.2.4 (http://www.R-project.org).

## Results

### Patient characteristics

The baseline characteristics of the patients are summarized in Table 1. A total of 727 patients with pathologically confirmed AGC were analyzed. The majority of patients were male (68.0%), and the median age at diagnosis of AGC was 60 years (range 26–94) years. All patients underwent radical gastrectomy; 6.9% were pathological stage I, 26.3% were stage II, 57.4% were stage III, and 9.5% were stage IV. This study included 63 stage IV patients who presented with enlarged paraaortic lymph nodes that were observed by either preoperative PET/CT or CT. These patients underwent radical surgical resection with a curative aim accompanied by paraaortic lymph node dissection. Regarding the WHO classification, 36.9% had WMD histology, 48.0% had PD histology, and the remaining 15.1% had SRC histology. When patients were classified according to the Lauren classification, 46.8% had intestinal-type AGC, 44.6% had diffuse type, and 8.7% had mixed type. Adjuvant chemotherapy was given in 88% of patients, excluding stage I patients (50 patients), those with poor performance after surgery (16 patients), and 21 patients who refused chemotherapy. All patients who had adjuvant chemotherapy received fluorouracil-based chemotherapy.

**Table 1:** Clinicopathological features and SUV_max_

Variables	Total (*n* = 727) *n* (%)	SUV_max_ Mean (SD)	SUV_max_ Median (range)	*P* value
Age (years)				
Median (range)	60.0 (26–94)			
Sex				
Male	494 (68.0)	7.8 ± 5.1	6.3 (1.7–39.1)	0.274
Female	233 (32.0)	7.4 ± 6.1	4.9 (1.3–42.6)	
Location of tumor				
Upper	144 (19.8)	7.6 ± 5.0	6.0 (1.3–28.6)	0.250
Middle	203 (27.9)	7.5 ± 5.6	5.9 (1.9–42.6)	
Lower	365 (50.2)	7.9 ± 5.7	6.1 (1.7–39.1)	
Whole	15 (2.1)	5.3 ± 2.5	4.4 (2.7–10.5)	
Histology (WHO)				
WMD	268 (36.9)	9.2 ± 6.1	7.8 (1.7–42.6)	< 0.001
PD	349 (48.0)	7.5 ± 5.3	5.6 (1.3–36.0)	
SRC	110 (15.1)	4.5 ± 1.9	4.1 (1.9–11.8)	
Histology (Lauren)				
Intestinal	340 (46.8)	9.2 ± 6.2	7.6 (1.7–42.6)	< 0.001
Diffuse	324 (44.6)	6.2 ± 4.0	4.6 (1.3–26.5)	
Mixed	63 (8.7)	7.6 ± 5.9	5.6 (2.2–28.6)	
Stage				
I	50 (6.9)	5.8 ± 3.8	4.3 (1.7–18.9)	0.043
II	191 (26.3)	8.3 ± 6.5	6.1 (2.1–42.6)	
III	417 (57.4)	7.7 ± 5.2	6.0 (1.9–36.0)	
IV	69 (9.5)	7.4 ± 4.6	5.9 (1.3–22.4)	
T stage				
T2	93 (12.8%)	6.0 ± 3.5	4.7 (1.7–18.9)	< 0.001
T3	206 (28.3)	8.9 ± 6.2	7.1 (2.0–39.1)	
T4	428 (58.9)	7.5 ± 5.3	5.7 (1.3–42.6)	
N stage				
N0	163 (22.4)	7.5 ± 6.3	4.9 (1.7–42.6)	0.427
N1	135 (18.6)	8.2 ± 6.0	7.0 (2.0–39.1)	
N2	129 (17.7)	8.0 ± 5.3	6.3 (1.9–29.9)	
N3	300 (41.3)	7.4 ± 4.8	5.9 (1.3–32.6)	

*WMD* adenocarcinoma well to moderately differentiated, *PD* adenocarcinoma poorly differentiated, *SRC* signet ring cell carcinoma

### SUV_max_ and histologic subtype

**Fig. 1:**
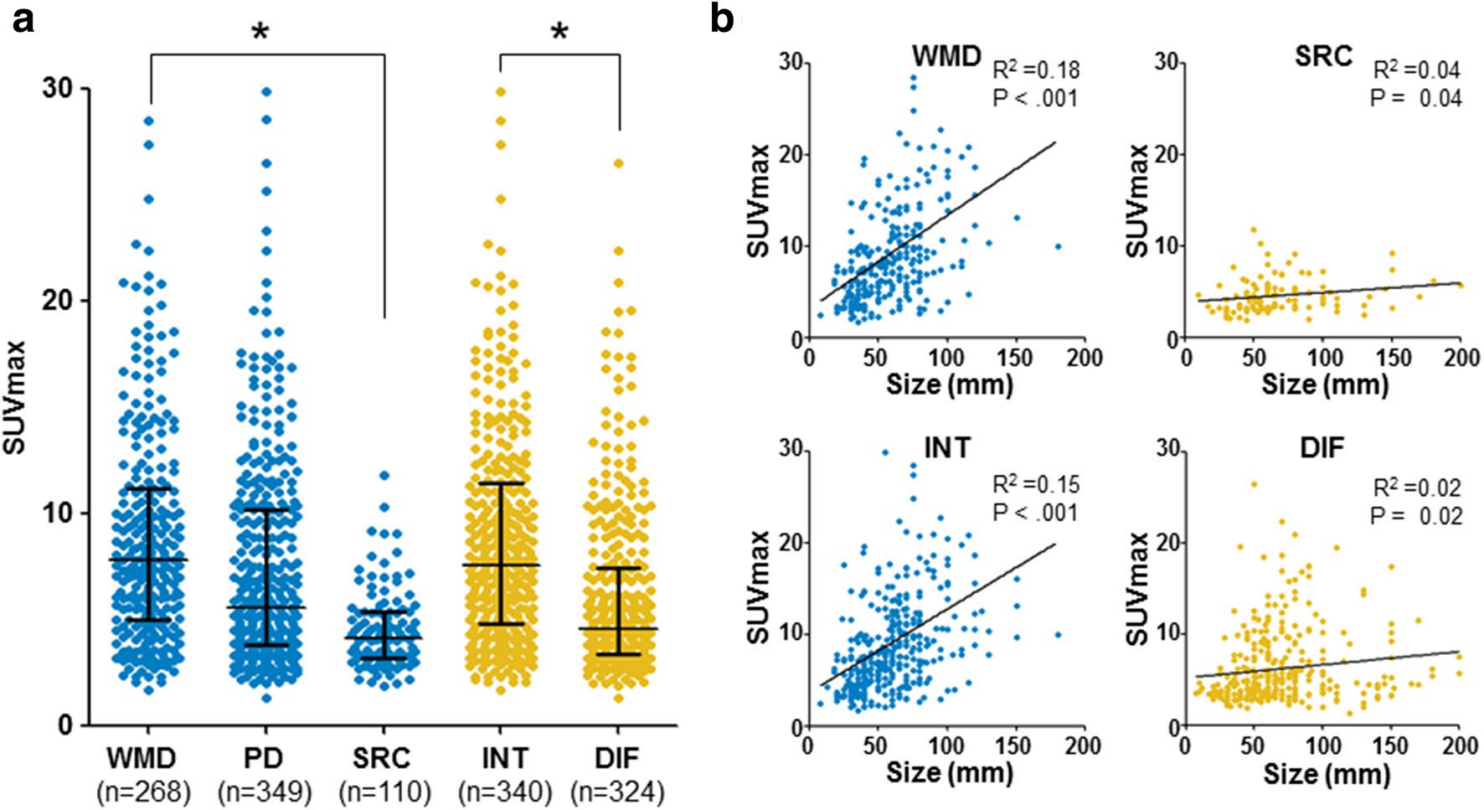
The preoperative ^18^F-FDG PET/CT SUVmax of advanced gastric cancer (AGC) according to histologic subtype. **a** The SUV_max_ correlates with the histologic type of AGC by both the WHO and Lauren classifications. **b** The SUV_max_ correlates well with the maximal size of the tumor in AGC with well to moderately differentiated (WMD) histology or intestinal type. *P < 0.05

This study only included patients with AGC, and 80% of all patients showed a positive ^18^F-FDG uptake (Supplementary Figure 1). In terms of WHO classification, 86% of WMD, 81% of PD, and 68% of SRC showed positive ^18^F-FDG uptake. According to the Lauren classification, 84% of intestinal type and 76% of diffuse type GC showed positive ^18^F-FDG uptake. In terms of stage, 72% of stage I, 75% of stage II, 82% of stage III, and 94% of stage IV showed positive ^18^F-FDG uptake. Table 1 shows SUV_max_ according to various clinicopathologic variables. Notably, SUV_max_ was significantly correlated with the histologic type of AGC by both the WHO and Lauren classifications (Fig. 1a). The mean SUV_max_ of AGC patients with SRC histology was 51% lower than that of AGC patients with WMD histology (4.5 ± 1.9 vs. 9.2 ± 6.1, *P* < 0.001). The majority of patients that had AGC with SRC histology had SUV_max_ less than 5, whereas the majority of patients that had AGC with WMD histology had SUV_max_ greater than 5. When the SUV_max_ was analyzed according to the Lauren classification, patients with diffuse-type AGC, which mostly had SRC histology, had 33% lower SUV_max_ than those with intestinal-type AGC, which mostly had WMD histology, (6.2 ± 4.0 vs. 9.2 ± 6.2, *P* < 0.001) (Fig. 1a). Moreover, the SUV_max_ also correlated with the progression of stage, especially T stage (*P* < 0.001), but not nodal (N) stage (*P* = 0.427). Intriguingly, the SUV_max_ correlated well with the maximal size of the tumor mass in AGC with WMD histology or intestinal type (Fig. 1b). However, the degree of correlation between SUV_max_ and maximal tumor size was relatively weak in AGC with SRC histology or diffuse type. Collectively, these findings demonstrate the distinct tumor biology of AGC with WMD or SRC histology, especially in terms of glucose metabolism.

### The prognostic impact of SUV_max_ according to histologic subtype

**Fig. 2:**
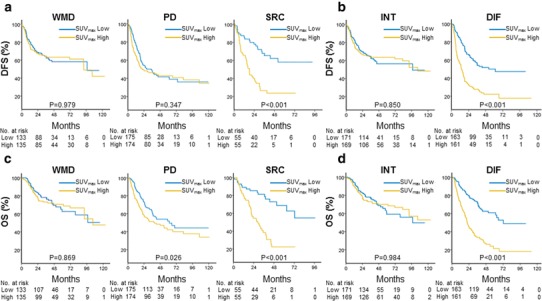
Kaplan–Meier survival curves comparing the high- and low-SUV_max_ groups in each histologic subtype. **a, b** High SUV_max_ only had a negative prognostic impact on disease-free survival (DFS) in AGC with SRC histology or diffuse type. **c, d** High SUV_max_ only had a negative prognostic impact on overall survival (OS) in AGC with SRC histology or diffuse type

With the median follow-up duration of 32.5 months, 357 (49%) patients recurred and 301 (49%) died. To evaluate the prognostic impact of each histologic subtype, the survival outcomes were compared between the high- and low-SUV_max_ groups. The cut-off value was the median SUV_max_ of each histologic group (Table 1). In terms of DFS, AGC patients with high SUV_max_ had significantly shorter DFS if they had diffuse-type AGC or SRC histology (*P* < 0.001 and *P* < 0.001, respectively), while there were no differences in the DFS of AGC patients with intestinal type or WMD histology (Fig. 2a, b). This was also true for OS; high SUV_max_ only had a negative prognostic impact in AGC with diffuse type or SRC histology (*P* < 0.001 and *P* < 0.001, respectively; Fig. 2c, d).

In the Cox proportional hazard model, which was adjusted for sex, age, T stage and N stage (Table 2), high SUV_max_ was also negatively correlated with DFS and OS in AGC patients with SRC histology (DFS: HR 2.26, *P* = 0.005; OS: HR 2.61, *P* = 0.003). Moreover, high SUV_max_ was negatively correlated with DFS and OS in AGC patients with diffuse-type AGC (DFS: HR 2.17, *P* < 0.001; OS: HR 2.47, *P* < 0.001). This negative prognostic impact was not observed in AGC patients with WMD histology or intestinal type. In addition, even when the Cox regression model was applied with the exception of 16 stage IV patients, high SUV_max_ was still a poor prognostic factor in SRC and diffuse-type gastric cancer (Supplementary Table 2).

**Table 2:** Multivariable cox regression analysis of SUV_max_ and its predictive impact on clinical outcomes according to histologic type

WHO classification		WMD (*n* = 268)		PD (*n* = 349)		SRC (*n* = 110)	
		AHR (95% CI)	*P* value	AHR (95% CI)		AHR (95% CI)	*P* value
DFS							
Sex	Female vs. male (ref)	0.97 (0.58–1.62)	0.916	0.94 (0.70–1.27)	0.686	0.78 (0.47–1.31)	0.354
Age	≥ 65 vs. < 65 years (ref)	1.47 (0.98–2.18)	0.060	1.47 (1.09–1.97)	0.011	1.43 (0.83–2.47)	0.198
T stage	T4 vs. T2 + T3 (ref)	3.18 (2.08–4.87)	< 0.001	2.54 (1.75–3.68)	< 0.001	5.44 (1.93–15.34)	0.001
N stage	N1 + N2 + N3 vs. N0 (ref)	1.99 (1.14–3.46)	0.015	3.12 (1.75–5.55)	< 0.001	2.25 (0.88–5.75)	0.090
SUV_max_	High vs. low (ref)	0.92 (0.62–1.37)	0.680	1.09 (0.81–1.45)	0.570	2.26 (1.28–4.00)	0.005
OS							
Sex	Female vs. male (ref)	0.95 (0.53–1.69)	0.854	1.00 (0.73–1.38)	1.000	0.86 (0.50–1.48)	0.574
Age	≥ 65 vs. < 65 years (ref)	1.99 (1.27–3.14)	0.003	1.73 (1.26–2.37)	0.001	1.98 (1.12–3.50)	0.018
T stage	T4 vs. T2 + T3 (ref)	3.13 (1.92–5.09)	< 0.001	2.87 (1.90–4.34)	< 0.001	3.68 (1.29–10.48)	0.015
N stage	N1 + N2 + N3 vs. N0 (ref)	1.58 (0.87–2.89)	0.135	3.04 (1.59–5.85)	< 0.001	2.22 (0.77–6.35)	0.138
SUV_max_	High vs. low (ref)	0.90 (0.57–1.41)	0.638	1.39 (1.01–1.90)	0.043	2.61 (1.39–4.91)	0.003

Lauren classification		Intestinal (*n* = 340)		Mixed (*n* = 63)		Diffuse (*n* = 324)	
		AHR (95% CI)	*P* value	AHR (95% CI)		AHR (95% CI)	*P* value
DFS							
Sex	Female vs. male (ref)	1.02 (0.66–1.59)	0.922	0.65 (0.29–1.42)	0.277	0.97 (0.72–1.29)	0.814
Age	≥ 65 vs. < 65 years (ref)	1.72 (1.21–2.44)	0.003	0.82 (0.38–1.77)	0.615	1.35 (1.00–1.83)	0.051
T stage	T4 vs. T2 + T3 (ref)	2.87 (1.96–4.20)	< 0.001	3.63 (1.25–10.59)	0.018	3.09 (2.04–4.67)	< 0.001
N stage	N1 + N2 + N3 vs. N0 (ref)	2.28 (1.37–3.81)	0.002	2.45 (0.67–8.92)	0.174	2.55 (1.44–4.53)	0.001
SUV_max_	High vs. low (ref)	0.80 (0.56–1.13)	0.202	0.69 (0.32–1.53)	0.363	2.17 (1.60–2.95)	< 0.001
OS							
Sex	Female vs. male (ref)	1.09 (0.67–1.76)	0.742	0.77 (0.34–1.76)	0.536	0.99 (0.72–1.35)	0.932
Age	≥ 65 vs. < 65 years (ref)	2.11 (1.42–3.14)	< 0.001	1.27 (0.57–2.84)	0.558	1.74 (1.26–2.41)	< 0.001
T stage	T4 vs. T2 + T3 (ref)	2.83 (1.84–4.37)	< 0.001	4.13 (1.24–13.78)	0.021	3.13 (1.99–4.93)	< 0.001
N stage	N1 + N2 + N3 vs. N0 (ref)	1.82 (1.05–3.14)	0.033	2.11 (0.57–7.81)	0.265	2.79 (1.41–5.52)	0.003
SUV_max_	High vs. low (ref)	0.79 (0.53–1.18)	0.251	0.63 (0.27–1.46)	0.280	2.47 (1.77–3.46)	< 0.001

*WMD* adenocarcinoma well to moderately differentiated, *PD* adenocarcinoma poorly differentiated, *SRC* signet ring cell carcinoma, *DFS* disease-free survival, *OS* overall survival, *AHR* adjusted hazard ratio, *CI* confidence interval, *ref* reference

To externally validate these findings, we also analyzed data from an independent institution. The same results were consistently observed in this validation cohort: (1) diffuse-type AGC with SRC histology had lower SUV_max_ compared with intestinal-type AGC with WMD histology (Table S1); and (2) higher preoperative SUV_max_ indicated poorer prognosis in AGC with SRC histology and diffuse type, but not in AGC patients with WMD histology and intestinal type (Fig. S2).

Taken together, these data confirmed that high SUV_max_ has an independent negative prognostic role in AGC patients with SRC or diffuse-type AGC.

### Prognostic implications of SUV_max_ as a continuous variable (p-spline curve)

**Fig. 3:**
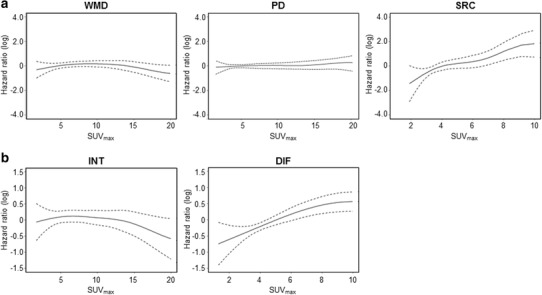
p-spline curves for DFS after adjusting for sex, age, and disease stage. The p-spline curves show a gradual increase in log HR as SUV_max_ rises only for SRC histology (**a**, right) and for diffuse type (**b**, right). There was no definite trend for WMD and PD histology (**a**, left) or intestinal type (**b**, left)

To further investigate the role of SUV_max_ as a continuous variable in survival analysis, p-spline curves for DFS were generated with the R program as described previously [15] after adjusting for sex, age, T stage and N stage (Fig. 3). The results were consistent with those from the Cox regression analysis with dichotomous variables. The p-spline curves showed a gradual increase in log HR as SUV_max_ rises only for SRC histology (Fig. 3a, right) and for diffuse type (Fig. 3b, right). There was no definite trend for WMD and PD histology (Fig. 3a, left) or intestinal type (Fig. 3b, left). This confirmed that SUV_max_ is a continuous variable that can predict DFS in AGC patients with SRC histology or diffuse-type AGC.

### Generating a predictive model for recurrence probability based on preoperative SUV_max_ in SRC or diffuse-type AGC

**Fig. 4:**
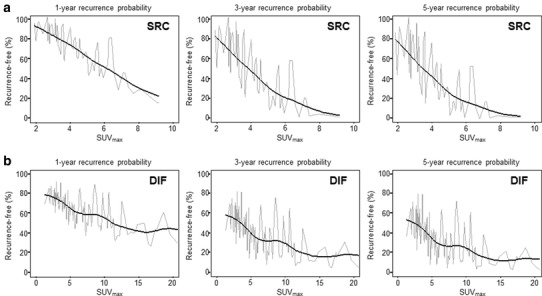
Predictive model for recurrence based on preoperative SUV_max_ in SRC (**a**) or diffuse-type AGC (**b**)

**Fig. 5:**
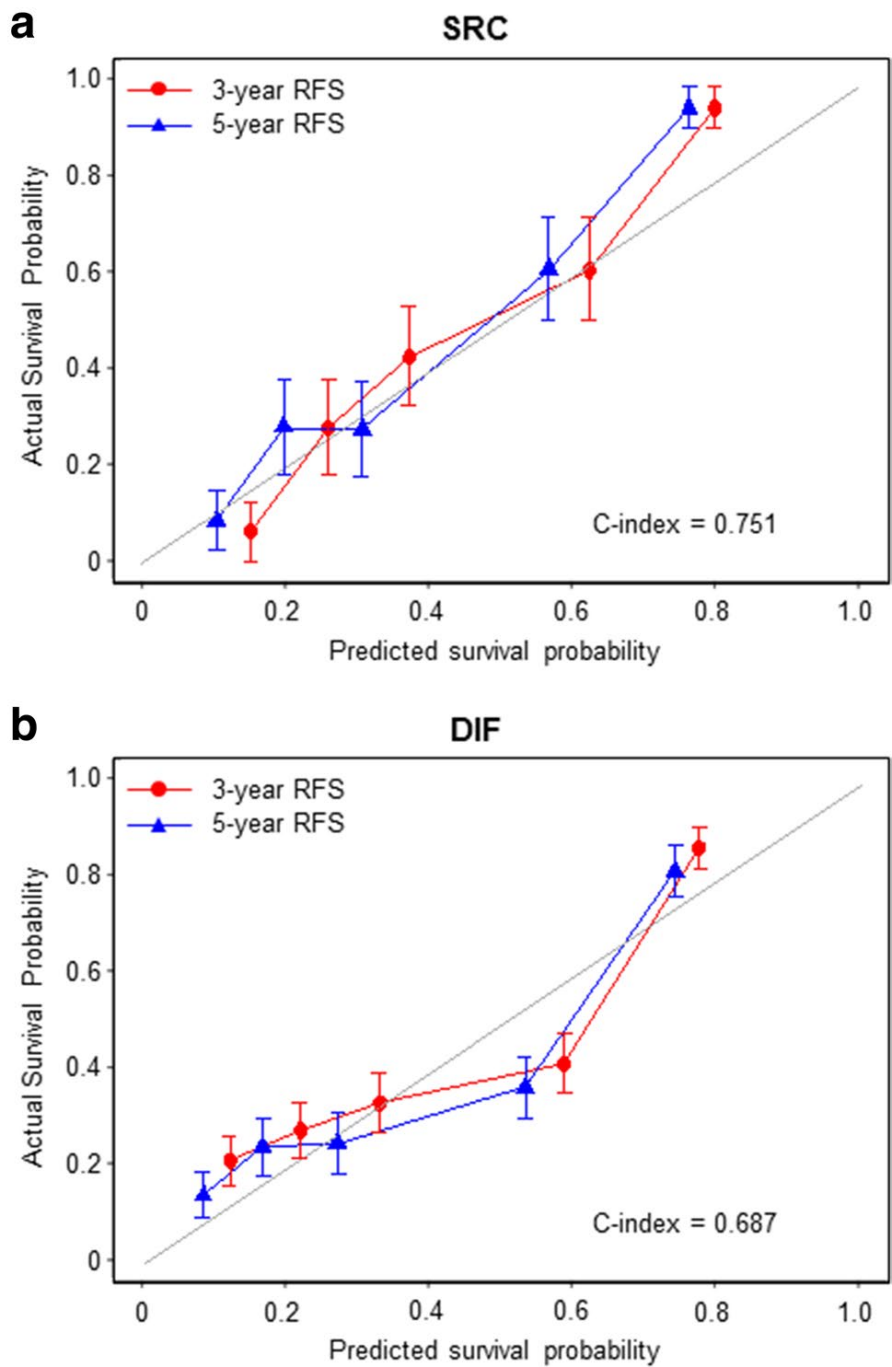
Calibration curves for the performance of predictive model in SRC (**a**) or diffuse-type AGC (**b**)

To predict recurrence after curative surgery more precisely for AGC, we tried to develop a novel predictive model based on preoperative SUV_max_. Recurrence-free probabilities at 1, 3, and 5 years were calculated for AGC with SRC histology (Fig. 4a) or diffuse type (Fig. 4b) after adjusting for sex, age, T stage and N stage. The RFS rate gradually decreased as SUV_max_ increased, and the 5-year RFS rate was less than 20% when the SUV_max_ was greater than 5. To evaluate the performance of our predictive model, we generated calibration curves (Fig. 5) that showed good agreement between the predicted and actual RFS; the bootstrap-corrected c-indices of the model were 0.751 (95% CI 0.675–0.827) for AGC with SRC histology and 0.687 (95% CI 0.644–0.730) for diffuse-type AGC. Thus, we were able to generate and internally validate our novel predictive model for recurrence in AGC with SRC histology or diffuse-type AGC.

## Discussion

Gastric cancer is increasingly recognized as a heterogeneous disease [11, 14, 16, 17]. Classically, it is classified according to its histology, i.e., as intestinal type or diffuse type [18]. Intestinal-type GC is more predominant in older people and in men, whereas diffuse-type GC is more frequently found in younger women. Recently, genomic data is widely utilized to develop molecular classification systems for GC. The TCGA Research Network proposed a classification system to distinguish GC into four subtypes: (1) Epstein–Barr virus (EBV)-positive, with the highest DNA methylation levels; (2) microsatellite instability (MSI), characterized by hypermutated tumors; (3) genomic stability (GS), which represents 20% of GC and comprises the majority of diffuse-type GC, has the most abundant CDH1 mutations and also shows increased RHOA mutations and CLDN18–ARHGAP fusions; and (4) chromosomal instability (CIN), which accounts for 50% of patients and is characterized by frequent TP53 mutations and high percentage of intestinal-type GC [16, 19].

However, current clinical practice does not take this heterogeneity into account; rather, GC is regarded as a single type of malignancy, and a one-size-fits-all approach is applied. Although some previous studies have shown that the SUV_max_ in PET/CT can differ markedly according to the histologic subtype, most studies still evaluate the prognostic impact of SUV_max_ by considering GC to be a single disease entity rather than categorizing it into the various histologic subtypes. Not surprisingly, this has resulted in inconsistencies between studies. Furthermore, despite the large number of studies that have already observed poor ^18^F-FDG PET uptake in patients with EGC only having mucosal or submucosal invasion, most studies still enroll patients with EGC. The largest preoperative study to date was reported by Lee et al., who evaluated the prognostic impact of PET/CT in 271 GC patients [8]. Because approximately half of the enrolled patients had EGC, 45% of the patients had no detectable ^18^F-FDG uptake, and so only the remaining 149 patients were available for further analysis. Consequently, the subgroup analysis was limited by the small sample size.

To overcome the limitations of previous studies, the present study prospectively collected data from more than 700 patients with AGC while excluding patients with EGC. Moreover, to avoid oversimplification, the patients were divided and analyzed according to their histologic subtypes. Furthermore, the prognostic impact of SUV_max_ was evaluated not only as a dichotomous variable as determined by the median SUV_max_ but also as a continuous variable by analyzing p-spline curves. As a result, we were able to reveal the distinct prognostic impact of SUV_max_ in ^18^F-FDG PET/CT according to histologic subtype.

First, diffuse-type AGC with SRC histology had lower SUV_max_ compared with intestinal-type AGC with WMD histology, which is in agreement with previous studies. Second, although the SUV_max_ of diffuse-type AGC was lower than that of intestinal type, it had a significant prognostic impact in terms of survival outcome. On the other hand, the SUV_max_ of intestinal-type AGC was more directly correlated with primary tumor size than diffuse-type AGC, but it did not have any prognostic impact. These finding provide a clue to explaining previous discrepancies regarding the prognostic impact of ^18^F-FDG PET/CT in GC patients. Finally, we established and validated a novel model that utilizes the preoperative SUV_max_ to predict tumor recurrence after surgery in patients with SRC or diffuse type, which may be a useful tool for clinical application.

Each histologic subtype of GC differs in its biology, especially in its metabolic profiles, which leads to different ^18^F-FDG uptake patterns [20]. Among the various histologic types of GC, SRC stands out as a unique subtype due to its distinct molecular and metabolic features. In terms of the glucose transporter GLUT-1, SRC is reported to express GLUT-1 at lower levels than WMD adenocarcinoma, leading to reduced ^18^F-FDG uptake [21, 22]. In addition, SRC has lower levels of the pyruvate kinase M2 isoform (PKM2) compared with other histologic subtypes; PKM2 is responsible for ATP production in the last step of glycolysis [12]. Furthermore, PKM2 expression is correlated with poor prognosis in SRC, while other subtypes are not. These metabolic characteristics help explain the different patterns and prognostic values of ^18^F-FDG PET/CT in different histologic subtypes of GC, and our data highlight the importance of dividing GC into different histologic subtypes before PET/CT analysis.

The main limitation of our study is the retrospective nature of data collection. Although we verified our findings in two independent cancer centers in Korea, more studies are needed to validate our findings in a prospective cohort. Especially, this study did not include patients who underwent preoperative treatment. Therefore, it is necessary to evaluate the role of PET/CT in Western patients who received preoperative treatment with other studies. In addition, this study only included patients with AGC, thus most patients received adjuvant chemotherapy. There are limitations in analysis of the effect of adjuvant chemotherapy on the prognostic remodeling. Second, volumetric PET parameters were not measured due to the large number of cases. Additional studies are needed to further evaluate the value of volumetric PET parameters rather than SUV_max_ for predicting clinical outcomes in intestinal-type AGC.

In conclusion, this study demonstrated the differential patterns and prognostic impact of preoperative PET/CT SUV_max_ in AGC according to histologic type. Although the SUV_max_ did not have significant prognostic impact in WMD- and intestinal-type AGC, higher preoperative SUV_max_ indicated poorer prognosis in SRC and diffuse-type AGC. Novel predictive models for recurrence probability can be provided based on the preoperative SUV_max_ in patients with SRC or diffuse-type AGC. To validate these findings, we are preparing a prospective trial. If the results of this study are confirmed in a prospective trial, SRC or diffuse-type gastric cancer patients with high SUV_max_ should be stratified in adjuvant chemotherapy. Ultimately, a clinical trial in which novel or more intensive therapy approaches are applied to SRC and diffuse-type AGC patients with high SUV_max_ should be performed.

## Electronic supplementary material

Below is the link to the electronic supplementary material.

Supplementary material 1 (DOC 169 KB)

Supplementary Figure 1. CONSORT diagram (TIF 75 KB)

Supplementary Figure 2. Kaplan–Meier survival curves of validation cohort comparing the high- and low SUV_max_ groups in each histologic subtype (TIF 129 KB)
