# Supplementary material for: The clinical implications of FDG-PET/CT differ according to histology in advanced gastric cancer
Source: Gastric Cancer. 2018 Jun 9;22(1):113–22. doi: 10.1007/s10120-018-0847-5 (PMC6314995; doi:10.1007/s10120-018-0847-5)
Supplement: Supplementary file 1 — Supplementary material 1 (DOC 169 KB) [file 10120_2018_847_MOESM1_ESM.doc]

**Supplementary Table 1.** Clinicopathological features and SUV_max_ of the validation cohort

| **Variables** | **Total (n = 173)**  **n (%)** | | **SUV_max_**  **Mean (SD)** | **SUV_max_**  **Median (range)** | **P*-value*** |
| --- | --- | --- | --- | --- | --- |
| **Age, years** | |  |  |  |  |
| Median (Range) | | 64.0 | (28-89) |  |  |
| **Sex** | |  |  |  |  |
| Male | | 123 (71.1) | 5.6 ± 3.5 | 4.2 (1.1-17.4) | 0.386 |
| Female | | 50 (28.9) | 6.1 ± 3.9 | 4.9 (2.5-18.7) |  |
| **Location of tumor** | |  |  |  |  |
| Upper | | 41 (23.7) | 5.9 ± 3.6 | 5.0 (2.5-15.5) | 0.819 |
| Middle | | 20 (11.6) | 5.0 ± 3.5 | 4.2 (1.1-16.9) |  |
| Lower | | 108 (62.4) | 5.8 ± 3.7 | 4.5 (2.5-18.7) |  |
| Whole | | 4 (2.3) | 5.5 ± 3.5 | 4.5 (2.5-10.5) |  |
| **Histology (WHO)** | |  |  |  |  |
| WMD | | 75 (43.4) | 5.9 ± 3.6 | 4.6 (2.5-18.7) | 0.065 |
| PD | | 65 (37.6) | 5.9 ± 3.9 | 4.3 (1.1-17.4) | (WMD vs. SRC) |
| SRC | | 20 (11.6) | 4.3 ± 1.9 | 4.1 (2.5-10.0) |
| **Histology (Lauren)** | |  |  |  |  |
| Intestinal | | 98 (56.6) | 6.2 ± 3.9 | 4.7 (2.5-18.7) | 0.050 |
| Diffuse | | 66 (38.2) | 5.1 ± 3.2 | 4.3 (1.1-16.9) | (Int. vs.  Diffuse) |
| Mixed | | 7 (4.0) | 5.7 ± 3.4 | 5.1 (2.5-12.4) |
| **Stage** | |  |  |  |  |
| I | | 27 (15.6) | 3.9 ± 2.0 | 3.0 (2.5-8.5) | 0.029 |
| II | | 50 (28.9) | 5.7 ± 3.9 | 4.2 (2.5-17.4) |  |
| III | | 89 (51.4) | 6.3 ± 3.5 | 5.1 (2.5-16.9) |  |
| IV | | 7 (4.0) | 5.7 ± 5.9 | 4.2 (1.1-18.7) |  |
| **T Stage** | |  |  |  |  |
| T2 | | 49 (28.3%) | 4.1 ± 2.4 | 3.3 (1.1-14.2) | 0.001 |
| T3 | | 65 (37.6) | 6.2 ± 3.8 | 4.8 (2.5-17.4) |  |
| T4 | | 59 (34.1) | 6.6 ± 3.9 | 5.0 (2.5-18.7) |  |
| **N Stage** | |  |  |  |  |
| N0 | | 50 (28.9) | 5.1 ± 3.5 | 3.5 (2.5-17.0) | 0.108 |
| N1 | | 27 (15.6) | 4.8 ± 3.6 | 3.9 (1.1-17.4) |  |
| N2 | | 32 (18.5) | 6.5 ± 3.9 | 4.9 (2.5-16.9) |  |
| N3 | | 64 (37.0) | 6.2 ± 3.5 | 5.0 (2.5-18.7) |  |
| Abbreviations: WMD, adenocarcinoma well to moderately differentiated; PD, adenocarcinoma poorly differentiated; SRC, signet ring cell carcinoma. | | | | |  |

**Supplementary Table 2.** Multivariable cox regression analysis of SUV_max_ and its predictive impact on clinical outcomes according to histologic type (except for stage IV patients).

| **WHO classification** | | **WMD (n = 247)** | | **PD (n = 309)** | | **SRC (n = 102)** | |
| --- | --- | --- | --- | --- | --- | --- | --- |
|  |  | AHR (95% CI) | P-value | AHR (95% CI) |  | AHR (95% CI) | P-value |
| **DFS** |  |  |  |  |  |  |  |
| Sex | Female *vs.* male (ref) | 0.92 (0.52-1.61) | 0.775 | 0.83 (0.58-1.17) | 0.298 | 0.75 (0.43-1.32) | 0.332 |
| Age | ≥ 65 *vs.*<65 years (ref) | 1.48 (0.95-2.29) | 0.077 | 1.64 (1.18-2.28) | 0.003 | 1.24 (0.67-2.31) | 0.488 |
| T stage | T3 *vs.* T2 (ref) | 0.95 (0.43-2.09) | 0.903 | 1.76 (0.66-4.66) | 0.256 | 1.24 (0.12-12.54) | 0.855 |
|  | T4 *vs.* T2 (ref) | 2.54 (1.21-5.32) | 0.013 | 2.86 (1.11-7.36) | 0.029 | 4.84 (0.63-37.26) | 0.129 |
| N stage | N1 *vs.* N0 (ref) | 1.28 (0.63-2.60) | 0.492 | 1.27 (0.60-2.68) | 0.517 | 1.64 (0.49-5.52) | 0.418 |
|  | N2 *vs.* N0 (ref) | 1.19 (0.57-2.47) | 0.643 | 2.53 (1.25-5.09) | 0.009 | 1.43 (0.44-4.68) | 0.545 |
|  | N3 *vs.* N0 (ref) | 2.67 (1.40-5.09) | 0.003 | 3.80 (2.01-7.19) | <0.001 | 2.32 (0.86-6.24) | 0.094 |
| Size | ≥ 5 *vs.* <5cm (ref) | 1.21 (0.71-2.07) | 0.469 | 1.35 (0.91-2.00) | 0.131 | 2.09 (1.03-4.25) | 0.041 |
| SUV_max_ | High *vs.* Low (ref) | 0.89 (0.58-1.38) | 0.898 | 1.01 (0.73-1.40) | 0.923 | 2.42 (1.32-4.43) | 0.004 |
| **OS** |  |  |  |  |  |  |  |
| Sex | Female *vs.* male (ref) | 0.86 (0.46-1.62) | 0.656 | 0.90 (0.61-1.31) | 0.588 | 0.82 (0.45-1.50) | 0.535 |
| Age | ≥ 65 *vs.*<65 years (ref) | 2.04 (1.24-3.34) | 0.005 | 2.10 (1.46-3.02) | <0.001 | 1.78 (0.95-3.34) | 0.070 |
| T stage | T3 *vs.* T2 (ref) | 1.14 (0.47-2.75) | 0.759 | 2.51 (0.74-8.57) | 0.139 | 0.97 (0.09-10.07) | 0.982 |
|  | T4 *vs.* T2 (ref) | 2.52 (1.10-5.79) | 0.028 | 4.35 (1.31-14.39) | 0.016 | 2.65 (0.33-21.05) | 0.355 |
| N stage | N1 *vs.* N0 (ref) | 0.97 (0.43-2.20) | 0.956 | 0.98 (0.40-2.35) | 0.966 | 1.85 (0.50-6.88) | 0.355 |
|  | N2 *vs.* N0 (ref) | 1.10 (0.49-2.48) | 0.814 | 2.78 (1.26-6.09) | 0.011 | 0.84 (0.20-3.55) | 0.817 |
|  | N3 *vs.* N0 (ref) | 2.72 (1.35-5.51) | 0.005 | 4.13 (2.00-8.53) | <0.001 | 2.30 (0.76-6.97) | 0.138 |
| Size | ≥ 5 *vs.* <5cm (ref) | 0.93 (0.51-1.67) | 0.816 | 1.05 (0.69-1.61) | 0.793 | 2.27 (1.03-5.02) | 0.041 |
| SUV_max_ | High *vs.* Low (ref) | 0.82 (0.50-1.33) | 0.429 | 1.31 (0.91-1.88) | 0.133 | 2.90 (1.47-5.70) | 0.002 |
| **Lauren classification** | | **Intestinal (n = 314)** | | **Mixed (n = 59)** | | **Diffuse (n = 285)** | |
|  |  | AHR (95% CI) | P-value | AHR (95% CI) |  | AHR (95% CI) | P-value |
| **DFS** |  |  |  |  |  |  |  |
| Sex | Female *vs.* male (ref) | 0.94 (0.57-1.53) | 0.810 | 0.65 (0.26-1.60) | 0.349 | 0.83 (0.60-1.15) | 0.278 |
| Age | ≥ 65 *vs.*<65 years (ref) | 1.81 (1.23-2.67) | 0.003 | 0.87 (0.36-2.10) | 0.767 | 1.52 (1.09-2.13) | 0.013 |
| T stage | T3 *vs.* T2 (ref) | 1.32 (0.64-2.71) | 0.450 | 7819.02 (0-*) | 0.935 | 1.31 (0.48-3.60) | 0.589 |
|  | T4 *vs.* T2 (ref) | 2.86 (1.42-5.75) | 0.003 | 23397.24 (0-*) | 0.928 | 2.75 (1.08-7.01) | 0.034 |
| N stage | N1 *vs.* N0 (ref) | 1.46 (0.77-2.76) | 0.243 | 0.63 (0.10-3.96) | 0.625 | 1.18 (0.56-2.49) | 0.659 |
|  | N2 *vs.* N0 (ref) | 1.51 (0.78-2.93) | 0.217 | 1.60 (0.36-6.96) | 0.531 | 2.13 (1.04-4.35) | 0.037 |
|  | N3 *vs.* N0 (ref) | 2.92 (1.63-5.25) | <0.001 | 3.06 (0.77-12.03) | 0.109 | 3.06 (1.63-5.72) | <0.001 |
| Size | ≥ 5 *vs.* <5cm (ref) | 1.04 (0.65-1.68) | 0.856 | 3.08 (1.00-9.51) | 0.050 | 1.44 (0.98-2.13) | 0.061 |
| SUV_max_ | High *vs.* Low (ref) | 0.76 (0.52-1.12) | 0.169 | 0.67 (0.28-1.58) | 0.364 | 2.10 (1.50-2.93) | <0.001 |
| **OS** |  |  |  |  |  |  |  |
| Sex | Female *vs.* male (ref) | 0.93 (0.54-1.60) | 0.814 | 0.73 (0.28-1.85) | 0.510 | 0.87 (0.61-1.25) | 0.460 |
| Age | ≥ 65 *vs.*<65 years (ref) | 2.29 (1.47-3.55) | <0.001 | 1.43 (0.56-3.61) | 0.445 | 2.10 (1.46-3.02) | <0.001 |
| T stage | T3 *vs.* T2 (ref) | 1.38 (0.61-3.08) | 0.430 | 11412.25 (0-*) | 0.931 | 1.81 (0.51-6.33) | 0.351 |
|  | T4 *vs.* T2 (ref) | 2.68 (1.23-5.84) | 0.013 | 2826.52 (0-*) | 0.925 | 3.65 (1.11-11.98) | 0.032 |
| N stage | N1 *vs.* N0 (ref) | 0.98 (0.47-2.07) | 0.975 | 0.34 (0.03-3.50) | 0.371 | 1.35 (0.57-3.22) | 0.489 |
|  | N2 *vs.* N0 (ref) | 1.32 (0.63-2.77) | 0.454 | 1.57 (0.36-6.88) | 0.545 | 2.21 (0.95-5.17) | 0.065 |
|  | N3 *vs.* N0 (ref) | 3.12 (1.65-5.90) | <0.001 | 2.55 (0.63-10.20) | 0.185 | 3.30 (1.56-6.99) | 0.002 |
| Size | ≥ 5 *vs.* <5cm (ref) | 0.75 (0.44-1.29) | 0.308 | 3.11 (0.97-9.95) | 0.055 | 1.25 (0.82-1.91) | 0.293 |
| SUV_max_ | High *vs.* Low (ref) | 0.73 (0.47-1.12) | 0.159 | 0.64 (0.25-1.58) | 0.336 | 2.50 (1.71-3.66) | <0.001 |

Abbreviations: WMD, adenocarcinoma well to moderately differentiated; PD, adenocarcinoma poorly differentiated; SRC, signet ring cell carcinoma; DFS, disease-free survival; OS, overall survival; AHR, adjusted hazard ratio; CI, conﬁdence interval; ref, reference; * >10000
