# Supplementary figures and images for: The clinical implications of FDG-PET/CT differ according to histology in advanced gastric cancer
Source: Gastric Cancer. 2018 Jun 9;22(1):113–22. doi: 10.1007/s10120-018-0847-5 (PMC6314995; doi:10.1007/s10120-018-0847-5)

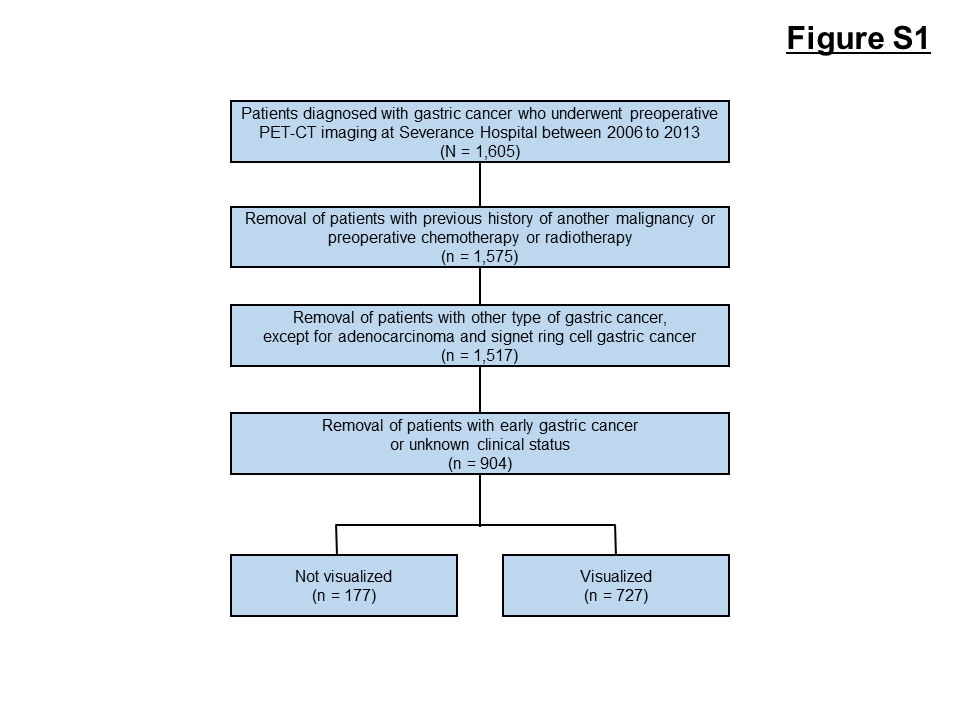

Supplement: Supplementary file 2 — Supplementary Figure 1. CONSORT diagram (TIF 75 KB) [file 10120_2018_847_MOESM2_ESM.tif]

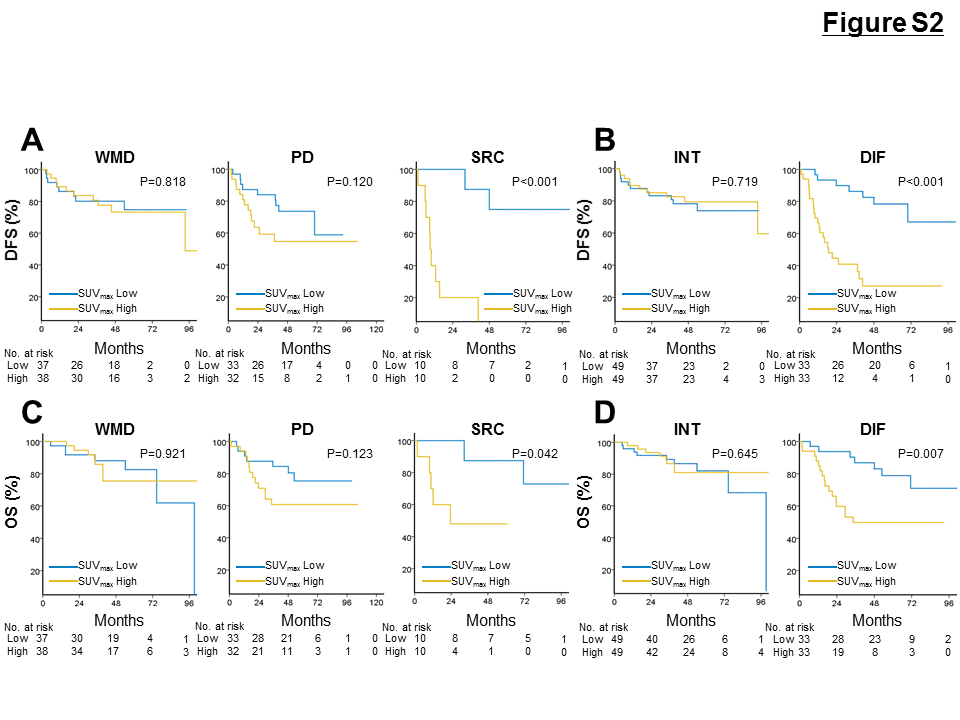

Supplement: Supplementary file 3 — Supplementary Figure 2. Kaplan–Meier survival curves of validation cohort comparing the high- and low SUVmax groups in each histologic subtype (TIF 129 KB) [file 10120_2018_847_MOESM3_ESM.tif]
